# Motivational disturbances in rodent models of neuropsychiatric disorders

**DOI:** 10.3389/fnbeh.2022.940672

**Published:** 2022-08-16

**Authors:** Tara Canonica, Ioannis Zalachoras

**Affiliations:** Department of Fundamental Neurosciences, University of Lausanne, Lausanne, Switzerland

**Keywords:** motivation, depression, stress, schizophrenia, parkinson’s disease, operant conditioning, dopamine

## Abstract

Motivated behavior is integral to the survival of individuals, continuously directing actions toward rewards or away from punishments. The orchestration of motivated behavior depends on interactions among different brain circuits, primarily within the dopaminergic system, that subserve the analysis of factors such as the effort necessary for obtaining the reward and the desirability of the reward. Impairments in motivated behavior accompany a wide range of neuropsychiatric disorders, decreasing the patients’ quality of life. Despite its importance, motivation is often overlooked as a parameter in neuropsychiatric disorders. Here, we review motivational impairments in rodent models of schizophrenia, depression, and Parkinson’s disease, focusing on studies investigating effort-related behavior in operant conditioning tasks and on pharmacological interventions targeting the dopaminergic system. Similar motivational disturbances accompany these conditions, suggesting that treatments aimed at ameliorating motivation levels may be beneficial for various neuropsychiatric disorders.

## Introduction

Motivation is a complex process that requires the constant monitoring of the environment and the orchestration of decisions regarding the allocation of effort and resources in relation to expected rewards or punishments (Chong et al., 2016). For instance, responses can be directed toward finding mating partners or food, and away from painful stimuli or stressful situations (Salamone et al., 2022). Hence, motivation involves a large number of behavioral programs related to reward-seeking, invigoration, and persistence (Salamone et al., 2022). As such, motivation is crucial for survival and well-being (Epstein et al., 2006; Crocker et al., 2013).

Given the variety of stimuli that elicit a motivational component, engaging a wide behavioral repertoire, it is unsurprising that motivational processes depend on the interaction and coordination of different brain circuits (Salamone et al., 2022). Nevertheless, one central neurobiological substrate of motivated behavior is the mesolimbic dopaminergic (DA-ergic) pathway, comprising DA-ergic projections from the Ventral Tegmental Area (VTA) to the Nucleus Accumbens (NAc) (Salamone John and Correa, 2012; Lammel et al., 2014; Salamone et al., 2015; Treadway and Salamone, 2022). Modulation of firing frequency of NAc-projecting DA neurons is associated with DA release in the NAc, which can in turn promote effort-related behavior via activation of DA receptors on medium spiny neurons (Soares-Cunha et al., 2016, 2018, 2020; Lohani et al., 2018).

Motivational disturbances accompany a wide range of neuropsychiatric conditions (Treadway and Zald, 2011; Treadway et al., 2012; Crocker et al., 2013; Zald and Treadway, 2017; Salamone et al., 2022). Despite the importance of motivation in daily life, it is often overlooked as a parameter in neuropsychiatric disorders, particularly with regard to therapeutic interventions (Pessiglione et al., 2018; Chang et al., 2019). Similarly, only few studies have investigated the occurrence and treatment of motivational dysfunction in animal models of neuropsychiatric disorders.

Motivation is commonly assessed in rodents with operant paradigms. In these tasks, animals are trained to perform a certain action (usually pressing a lever or nosepoking a sensor) to acquire a palatable reward (Malkki et al., 2010). Fixed ratio (FR) schedules indicate that a fixed number of responses is required to trigger the delivery of a reward, whereas progressive ratio (PR) schedules indicate that for every subsequent reward, a progressively higher number of responses is needed. Several aspects of effort-related behavior can be measured in operant tasks, such as the number of responses, the number of obtained rewards, or the breakpoint (i.e., last completed ratio in PR schedules) (Haluk and Wickman, 2010).

Here we provide a brief overview on motivational disturbances investigated with operant paradigms in genetic, pharmacological, and environmental rodent models of schizophrenia, depression, and Parkinsons’s disease, focusing on the pivotal role of the DA system in effort-related behavior. Our aim is to highlight the significance of motivational disturbances in these three groups of neuropsychiatric disorders and to prompt a more widespread investigation of therapeutic interventions targeting motivation levels.

## Schizophrenia

Schizophrenia is a psychiatric disorder with a prevalence of approximately 1% worldwide. It is characterized by alterations of thought, perception, and affect, such as cognitive deficits, delusions, hallucinations, apathy, and anhedonia (Millard et al., 2022). Indeed, it is also accompanied by deficits in reward processing and motivation (Barch et al., 2016). These include increased sedentary behavior and unwillingness to engage in activities that necessitate the exertion of high effort (Yang et al., 2020b), corresponding to the negative symptoms cluster of schizophrenia (Markou et al., 2013). At least partially, these deficits may be related to alterations of DA circuits (Sotoyama et al., 2021). Animal models of schizophrenia recapitulate these motivational deficits, together with other landmark features of the disorder (Ward et al., 2015).

There are schizophrenia rodent models that use manipulations during early development, such as neonatal lesions, pharmacological treatments, exposure to stress, or rearing in isolation. Other models use pharmacological manipulations in adult animals, targeting DA signaling or NMDA receptor signaling. Genetic models tackle different cellular pathways linked to schizophrenia, such as DISC1 knockout mice, 22q11.2 deletion models, conditional neuregulin 1 and ErbB4 knockout mice, dysbindin mutant mice, reelin 1-deficient mice, GAD67 knockdown mice (Kolata et al., 2018), or different humanized mouse models (Winship et al., 2018).

Despite their heterogeneity, motivation deficits accompany schizophrenia features in several models (Table 1), as for example in the humanized catechol-O-methyltrasnferase (COMT) variants transgenic model. COMT is an enzyme involved in the degradation of catecholamines, including DA, whose metabolization rate depends on Val/Met polymorphism. The Val allele codes for a faster COMT enzyme, resulting in lower DA input to postsynaptic neurons, while the Met allele results in slower DA degradation rates (Chen et al., 2004). In an operant task, mice carrying the Val allele performed fewer responses in FR4 sessions than mice carrying the Met allele. Val carrier mice were also more inclined to favor consumption of choice food concurrently available than to exert effort to obtain a more palatable reward (Yang et al., 2020a).

**TABLE 1:** Summary of the reviewed studies investigating motivation with operant conditioning tasks in rodent models of schizophrenia, depression, and PD, and the investigated pharmacological treatments to ameliorate motivation deficits.

Disorder	Species	Model	Motivated behavior affected	Treatment	Mechanism	Amelioration	References
Schizophrenia	Mice	Human COMT158 Met/Val	FR4 ↓				Yang et al., 2020b
		DN-DISC1	PR ↓				Johnson Alexander et al., 2013; Yang et al., 2020a
		Gpr139−/−	FR5 ↓				Johnson Alexander et al., 2013; Dao et al., 2022
		Gad1 cKO	PR =				Kolata et al., 2018
		Striatum D2R overexpression	PR ↓	Doxycycline	D2R normalization	Yes	Kellendonk et al., 2006; Drew et al., 2007
		NAc D2R overexpression	PR ↑				Drew et al., 2007; Gallo et al., 2018
	Rats	Perinatal PCP treatment	PR5 =				Wiley and Compton, 2004; Amitai et al., 2019
		Repeated PCP treatment	PR ↑				Gallo et al., 2018; Amitai et al., 2019
		Isolation rearing	PR =				Gallo et al., 2018; Amitai et al., 2019
Depression	Mice	Chronic CORT treatment	PR ↓	Sulpiride	D2R antagonist	No	Olausson et al., 2013; Peng et al., 2021
		Acute LPS treatment	FR1, FR10 ↓				Remus and Dantzer, 2016; Vichaya et al., 2019
		Chronic social defeat	PR ↓	Agomelatine	MT1/MT2 agonist, 5-HT 2C antagonist	Yes	Bergamini et al., 2016a; Wang et al., 2017
	Rats	Chronic CORT treatment	PR ↓	Amitriptyline	non-SSRI	Yes	Chenu et al., 2013; Olausson et al., 2013
		Acute IL-1β treatment	FR5 ↓	CT-005404	DAT inhibitor	Yes	Vichaya et al., 2019; Rotolo et al., 2021
		Chronic restraint stress	FR1, FR2, FR4 ↓				Tye et al., 2012; Xu et al., 2017
		Chronic unpredictable mild stress	PR =				Barr and Phillips, 1998; Wanat et al., 2013
PD	Rats	Bilateral 6-OHDA SNpc lesion	FR1 ↓	Ropinirole	D2/D3R agonist	Yes	Duty and Jenner, 2011; Drui et al., 2014
				L-dopa	DA precursor	No	
				Citaloparm	SSRI	No	
		Bilateral 6-OHDA mVTA lesion	FR1 =				Duty and Jenner, 2011; Drui et al., 2014
		Bilateral 6-OHDA SNpc lesion	FR1 ↓	Pramipexole	D2/D3R agonist	Yes	Favier et al., 2014; Favier et al., 2017
		Bilateral 6-OHDA SNpc lesion	FR1 ↓	SKF-38393	D1R agonist	No	Carnicella et al., 2014; Drui et al., 2014
				Sumanirole	D2R agonist	No	
				PD-128907	D3R agonist	Yes	
		Bilateral 6-OHDA SNpc lesion	FR1 ↓				Carnicella et al., 2014; Favier et al., 2017
		Bilateral 6-OHDA SNpc lesion and subthalamic DBS	FR1 ↓	Pramipexole	D2/D3R agonist	Yes	Martinez-Fernandez et al., 2016; Vachez et al., 2020

↓ indicates reduced performance; ↑ indicates increased performance; = indicates no change in performance.

5-HT 2C, serotonergic; 6-OHDA, 6-hydroxydopamine; cKO, conditional Knockout; COMT, catechol-O-methyltrasnferase; CORT, corticosterone; D1/2/3R, dopamine receptor 1/2/3; DA, dopamine; DAT, dopamine transporter; DBS, Deep Brain Stimulation; FR, Fixed Ratio; Gad1, Glutamate decarboxylase 1; Gpr139, G Protein-Coupled Receptor 139; LPS, lipopolysaccharide; Met, Methionine; MT1/MT2, melatonergic; NAc, Nucleus Accumbens; PCP, phencyclidine; PD, Parkinson’s disease; PR, Progressive Ratio; SNpc, Substantia Nigra pars compacta; SSRI, Selective Serotonin Reuptake Inhibitor; Val, Valine.

Similar findings have been noted in other genetic models of schizophrenia altering DA signaling. For example, mice carrying a Dominant negative-DISC1 (DN-DISC1) mutation, a gene coding for a scaffold protein involved in the DA system, displayed impaired PR performance, in addition to impairments in reversal learning (Johnson Alexander et al., 2013). Respectively, a mouse model of altered DA-ergic signaling lacking the orphan receptor GPR139 exhibited schizophrenia-like features such as deficits in sensorimotor integration, social behavior, object and conspecific recognition, together with impairments in an FR5 operant conditioning task. GPR139 has been shown to inhibit D2 receptor-dependent signaling and in fact, treatment with the D2 receptor antagonist haloperidol in GPR139 KO mice ameliorated several of the schizophrenia-related behavioral deficits (Dao et al., 2022). Although operant behavior following haloperidol treatment was not assessed, these data suggest that normalization of DA signaling may also ameliorate motivational deficits in schizophrenia.

In a transgenic model of selective overexpression of D2 receptors in the striatum, cognitive features of schizophrenia such as working memory and behavioral flexibility impairments, were accompanied by motivational deficits in an operant timing task and in a PR schedule, without affecting passive sucrose preference (Kellendonk et al., 2006; Drew et al., 2007). Interestingly, viral-mediated D2 receptor overexpression in the NAc targeting specifically D2 receptor-expressing neurons, produced the opposite effect, with animals displaying increased motivation and PR performance (Gallo et al., 2018). Although the overexpression techniques differ substantially between the two animal models, these findings stress the critical role of D2 receptors in operant performance, with a critical distinction in the function of D2 receptors in striatum and NAc.

Nevertheless, it must be noted that motivational disturbances are not always observed in rodent models of schizophrenia. For instance, no effect on motivation was observed in a model of isolation rearing (Amitai et al., 2019), in a model of perinatal treatment with phencyclidine (Wiley and Compton, 2004), and in a model of GABA reduction in the cortex and the hippocampus (Kolata et al., 2018). Discrepant findings suggest differences between models in the extent to which they affect circuits underlying motivation, whereby motivation deficits may not always be recapitulated along other features of schizophrenia.

## Depression and stress-related psychopathology

Several adaptations are activated in response to stress, both in the brain and the periphery. If stress exposure is short and mild, brain functioning usually returns to baseline levels. However, prolonged stress exposure may result in the development of stress-related psychiatric disorders, such as depression (Korte et al., 2005; McEwen and Akil, 2020). Motivational disturbances characterize this type of disorders, manifesting as loss of interest or pleasure in carrying out enjoyable activities. Stress exposure and depression have been associated to imbalances in monoamine neurotransmitters, including DA (Pizzagalli, 2014). Even acute stress exposure can induce functional changes in DA-ergic circuits, along with impaired reward-seeking behavior (Lowes et al., 2021). Such effects seem mediated by DA or GABAergic neurons of the VTA and by corticotropin-releasing hormone signaling (Lemos et al., 2012, 2019; Friedman et al., 2014; Holly et al., 2015).

Rodent models of depression recreate motivational deficits (Table 1) and other depression-like symptoms, such as anhedonia (Treadway et al., 2012; Crocker et al., 2013; Pizzagalli, 2014). Chronic stress exposure is commonly used to model depression in rodents, with models employing chronic unpredictable mild stress (Tye et al., 2012), chronic restraint stress (Xu et al., 2017), or chronic social defeat (Chaudhury et al., 2012). Other rodent models of depression include pharmacological manipulations (e.g., glucocorticoids treatment), genetic (e.g., stress-resilient or stress-susceptible lines) or transgenic models (e.g., 5-HT-related mutations) (Wang et al., 2017).

In a chronic restraint stress model, rats were trained to press a lever in an FR1 operant task either prior to or following a 28-day restraining period (Xu et al., 2017). Predictably, stress exposure prior to operant training produced motivation deficits in a series of reward-related learning tasks, including acquisition deficits of the FR1 schedule. In contrast, animals that were trained following the restraining period did not show FR1 impairments in a maintenance test but performed worse than controls when higher response rates were assessed (FR2 and 4). Chronic restraint stress can thus produce motivation deficits disrupting basic effort-related behavior and also future performance in more effort-demanding tasks.

Similarly, in a chronic social defeat model, mice performed fewer responses and obtained less rewards than controls in a PR test, along with cognitive deficits in a reversal learning paradigm and decreased appetitive behavior (Bergamini et al., 2016a). Oral treatment with the antidepressant agomelatine attenuated the stress-induced effects on motivated behavior. Agomelatine is a melatonergic agonist (MT1/MT2) and a serotonergic antagonist (5-HT 2C), and its therapeutic effects on motivation may be mediated by 5-HT 2C-expressing GABA interneurons in the VTA projecting onto DA neurons (Eberle-Wang et al., 1997). Agomelatine has in fact been shown to increase activity of DA neurons in the VTA (Chenu et al., 2013).

Since the effects of stress in the brain are largely regulated by glucocorticoids, a further way to model depression is repeated treatment with glucocorticoids. Rats treated with corticosterone in the drinking water for 14 days showed decreased performance in a PR task 21 days after the last treatment dose (Olausson et al., 2013). Corticosterone treatment in conjunction with the antidepressant amitriptyline, a reuptake inhibitor leading to enhanced monoamine neurotransmission, prevented deficits in operant behavior, suggesting again a common base for depressive and motivational symptoms.

In addition to producing motivation deficits, a 7-day corticosterone treatment has been found to also alter VTA DA-ergic activity, decreasing excitatory synaptic transmission (Peng et al., 2021). Intra-VTA administration of the D2 receptor antagonist sulpiride was found to restore excitatory transmission in the VTA but failed to rescue operant performance in a PR test, delineating a complex relationship between DA-ergic VTA activity and effort-related behavior.

Finally, evidence from several studies established a link between immune responses and depression, leading to inflammation-induced models of depression (Maes, 1995; Berk et al., 2013; Remus and Dantzer, 2016; Brás et al., 2022). Mice treated with lipopolysaccharide displayed depressive-like symptoms and motivational deficits in an operant test, both for obtaining grain pellets with an FR1 schedule and for a preferred chocolate reward with an FR10 schedule (Vichaya et al., 2019). Similarly, systemic treatment with IL-1β, a proinflammatory cytokine with a modulatory effect on monoamine synthesis and transmission, led to impairments in an FR5 paradigm (Rotolo et al., 2021). Interestingly, oral treatment with the DA transport inhibitor CT-005404 restored normal lever presses levels in IL-1β-treated mice and increased them above baseline levels in non-treated animals. The treatment was also found to increase extracellular DA levels in the NAc, highlighting again the key role of DA levels in mediating effort-related behavior.

Although models of depression are commonly based on chronic stress exposure, acute stress can be sufficient to induce motivational disturbances (Shafiei et al., 2012; Wanat et al., 2013; Bryce and Floresco, 2016). This relationship is however not always observed, even in chronic stress models (Phillips and Barr, 1997; Barr and Phillips, 1998). Differences may be related to methodological factors, such as animal species and strain, stress protocol, reward type, or individual differences in behavioral traits, as observed for example in stress-resilient vs. stress-susceptible rats (Martis et al., 2018; Zalachoras et al., 2022).

## Parkinson’s disease

Parkinson’s disease (PD) is a neurodegenerative disorder characterized by motor impairments (Poewe et al., 2017). PD results from the progressive death of DA cells in the substantia nigra pars compacta (SNpc), which provide the main DA-ergic input within the nigrostriatal pathway. Given the key role of DA in driving effort-directed behavior, it is not surprising that individuals with PD frequently display motivational deficits, with about one-third reporting apathy and depression (Aarsland et al., 2012).

In animal models of PD, loss of DA innervation is usually recreated via local administration of DA-specific neurotoxic agents [e.g., 6-hydroxydopamine (6-OHDA) or 1-methyl-4phenyl-1,2,3,6-tetrahydropyridine (MPTP)] but there are also models based on genetic manipulations (e.g., α-synuclein-related transgenic alterations) and on exposure to environmental risk factors (e.g., pesticides) (Duty and Jenner, 2011).

The exact mechanisms underlying neuropsychiatric symptoms in PD are unclear, particularly with regard to motivational dysfunction. Addressing motivation in PD is a complex matter since motivational deficits can arise as a by-product of motor deficits hindering the execution of goal-directed behavior, rather than representing a symptom *per se*. At present, only few studies have investigated effort-related behavior in rodent models of PD, all in lesion models (Table 1). Such studies successfully preserved motor function by performing only partial SNpc lesions, allowing to assess non-motor symptoms of pre-clinical PD.

For example, rats with partial 6-OHDA lesions in the bilateral SNpc, presented various neuropsychiatric symptoms while retaining motor performance (Drui et al., 2014). SNpc-lesioned rats displayed motivation deficits in an operant task, earning less rewards than non-lesioned rats in FR1 sessions. This deficit was not observed following lesions of the VTA, implicating DA fibers originating specifically from the SNpc in the pathogenesis of motivational disturbances in PD, although VTA DA neurons may play a role in higher FRs or PR tasks. SNpc-lesioned animals also displayed apathy- and depression-like symptoms, including lower preference for food-paired places, increased immobility rates in the forced swim test, and reduced time engaging in social interactions.

Partial nigrostriatal denervation has been repeatedly found to spare motor performance while producing motivational impairments in operant tests (Carnicella et al., 2014; Favier et al., 2017). Importantly, preference for sucrose consumption in passive choice tests is unaffected by SNpc lesions, suggesting a specific motivational deficit in the drive to work for rewards in PD and not in the perception of reward value.

Further evidence from PD rodent models indicates that in addition to the nigrostriatal pathway (Carnicella et al., 2014; Favier et al., 2014), mesolimbic DA pathways can also take part in modulating motivational states in PD (Lelos et al., 2016). Non-DA systems (i.e., noradrenergic, serotonergic, and cholinergic) are also relevant for motivated behavior, mediating depression-like symptoms in PD rodent models (Winter et al., 2007; Hui et al., 2014; Faggiani et al., 2015).

Motor symptoms of PD are generally treated pharmacologically with levodopa and DA agonists to enhance DA levels (Poewe et al., 2017). These treatments have been shown to rescue not only motor symptoms but also neuropsychiatric symptoms, including motivational deficits, both in PD patients and animal models (Carnicella et al., 2014; Drui et al., 2014). For example, chronic intraperitoneal administration of the D2/D3 receptor agonists ropinirole or pramipexole fully reverted operant deficits in SNpc-lesioned rats, with deficits reappearing once the treatment was suspended (Drui et al., 2014; Favier et al., 2014). A further study investigated specifically the efficacy of D1, D2, and D3 receptor agonists in SNpc-lesioned rats (Carnicella et al., 2014). Although anxiety- and depression-like symptoms were ameliorated by treatment with any of the three DA receptor agonists, effort-related behavior was rescued only by treatment with a D3 receptor agonist.

Supporting a key role of D3 receptors and of the SNpc in motivational dysfunction in PD, autoradiography studies showed that following SNpc lesions, D1 and D2 receptor levels remain unaltered, while D3 receptors are specifically down-regulated (Favier et al., 2017).

Besides low motivation levels due to DA loss, PD also frequently presents with motivational disturbances of the opposite valence as adverse effects of antiparkinsonian medications. In fact, prolonged DA replacement therapy, especially with DA agonists, has been associated with higher prevalence rates of impulse control disorders, whereby individuals cannot resist the urge to perform reward-based activities repeatedly, despite potentially detrimental consequences (e.g., compulsive gambling, eating, shopping, sexual behavior) (Weintraub et al., 2010). Indeed, analogous side effects of DA medication have also been observed in rodent models of PD (Engeln et al., 2016; Dardou et al., 2017; Jiménez-Urbieta et al., 2019). Although impulsive phenotypes lie outside the scope of this review, their occurrence in PD corroborates the importance of well-balanced DA levels to adequately energize goal-directed behavior.

In addition to pharmacological therapies, motor symptoms of PD can also be treated with Deep Brain Stimulation (DBS), a surgical procedure used to implant electrical stimulators in target brain regions to compensate for compromised DA activity (Benabid et al., 2009). Although DBS has been found to greatly improve motor function, it is also associated with aggravation of neuropsychiatric symptoms, primarily apathy and depression scores (Castrioto et al., 2014; Martinez-Fernandez et al., 2016). Indeed, a recent study using wireless microstimulators in freely moving rats found that subthalamic DBS exacerbated FR1 motivational deficits in rats with partial SNpc lesions and produced motivational deficits in non-lesioned rats (Vachez et al., 2020).

Overall, findings on PD reiterate the complexity and the necessity of finely-tuned DA levels in the regulation of motivational states, highlighting the key role of the SNpc and in particular of D3 receptors.

## Discussion

Despite arising from different biological, genetic, and environmental factors, motivational deficits accompany a wide range of neuropsychiatric disorders. Motivational deficits aggravate the patients’ quality of life (Fervaha et al., 2014), but this aspect is often overlooked, both in clinical assessment and therapeutic interventions (Pessiglione et al., 2018), as well as in animal studies modeling such disorders (Ward et al., 2011). Here, we aimed to give a brief overview of motivational deficits recapitulated in rodent models of common neuropsychiatric disorders.

The presence of motivational deficits in rodent models of schizophrenia, depression, and PD is not surprising. Indeed, several of the models directly tackle DA-ergic circuits known to underlie motivation, altering expression levels of DA receptors, DA uptake, or DA neuron firing. These alterations either mimic the human condition (e.g., DISC1 expression or SNpc degeneration), occur spontaneously (e.g., high DA firing in animals susceptible to stress), or are due to clinically relevant environmental factors (e.g., chronic stress inducing functional NAc alterations). Importantly, rodent models of schizophrenia, depression, and PD display both motivational deficits and other symptoms characteristic of the given disorder, suggesting that motivational disturbances are a core part of these human conditions.

Nevertheless, interpreting effort-related performance in rodent models of neuropsychiatric disorders can be complex. In line with the disorders, such models also display anhedonia, cognitive deficits, and motor impairments, which are difficult to distinguish from pure motivational disturbances. The contribution of such phenotypes to the lack of motivation to exert effort for obtaining and consuming rewards is often unclear. For example, in operant-conditioning tasks, animals may fail to initiate trials or respond less accurately but not necessarily due to lower motivation to obtain the reward (Bergamini et al., 2016b). Respectively, higher perseverance in attempts to obtain rewards may reflect increased motivation but might also indicate a cognitive inability to adjust to the task’s demands (Millar et al., 2017). Moreover, individual differences in anxiety or other behavioral traits may also affect performance (Lozano-Montes et al., 2019; Zalachoras et al., 2022).

Recently, some studies have employed touchscreen-based operant conditioning tasks to evaluate motivation levels in rodents (Phillips et al., 2017, 2018). In addition to assessing effort-related behavior, such tasks also allow assessment of multiple psychological domains in the same animal, using the same apparatus. The study of motivational disturbances may thus benefit from such tasks, as operant performance may be correlated with other relevant variables (e.g., working memory), allowing a systematic identification and measurement of potential confounds.

Despite its limitations, the investigation of motivational deficits in animal models of neuropsychiatric disorders represents a promising area of study. First, it may add further translational value to the models and a better understanding of the disorder’s pathophysiology. Second, it may play a key role in the development of new therapeutic interventions. Indeed, motivational deficits are critical predictors of functional outcomes in psychopathologies (Fervaha et al., 2014, 2016; Chang et al., 2020). For example, in individuals with schizophrenia, apathy scores were more strongly linked to functional impairment than cognitive deficits (Konstantakopoulos et al., 2011). Therapeutic group interventions targeting motivational deficits in individuals affected by schizophrenia or major depression have also proven effective in alleviating the disease burden, leading to positive outcomes such as increased engagement in meaningful activities, higher levels of confidence, and better mood (Thonon et al., 2021).

Finally, targeting motivational disturbances may improve the prognosis of neuropsychiatric disorders by prompting an earlier diagnosis. In fact, signs such as apathy and depression precede the occurrence of motor symptoms and the diagnosis of PD by several years (Schapira et al., 2017). Understanding the emergence and the progression of motivational deficits that characterize neuropsychiatric disorders could help identify individuals at highest risk and promote an earlier start of therapeutic interventions. Because similar motivational disturbances are observed in different neuropsychiatric disorders, the investigation of motivation levels and their treatment is likely to benefit various conditions.

## Author contributions

Both authors listed have made a substantial, direct, and intellectual contribution to the work, and approved it for publication.
