# Erratum: Motivational disturbances in rodent models of neuropsychiatric disorders

**DOI:** 10.3389/fnbeh.2023.1178215

**Published:** 2023-03-13

**Authors:** Frontiers Production Office

**Affiliations:** Frontiers Media SA, Lausanne, Switzerland

**Keywords:** motivation, depression, stress, schizophrenia, parkinson's disease, operant conditioning, dopamine

An omission to the funding section of the original article was made in error. The following sentence has been added: “Open access funding was provided by the University of Lausanne.”

The original article has been updated.

